# Thoracic Outlet Syndrome Case Report: Appropriate Diagnosis Can Expedite Patient Treatment and Prevent Negative Outcomes

**DOI:** 10.5811/cpcem.35488

**Published:** 2025-04-30

**Authors:** Hunter Triplett, Victoria Winter, Brandon Leary, Alexis Lee, Kathryn Sulkowski, Eugene Kang

**Affiliations:** * Kirk Kerkorian School of Medicine at University of Nevada Las Vegas, Las Vegas, Nevada; † Saint Mary’s College of California. Moraga, California; ‡ United States Air Force/UNLV School of Medicine. Las Vegas, Nevada

**Keywords:** case report, thoracic outlet syndrome, emergency medicine

## Abstract

**Introduction:**

Thoracic outlet syndrome (TOS) is a diagnosis classifying upper extremity symptoms caused by compression of the neurogenic and vascular structures between the clavicle and first rib. It is important to promptly decompress these structures to prevent long-term deficits and poor patient outcomes. However, TOS often presents in unique ways with substantial symptom variance, making it difficult to identify, diagnose, and promptly treat. Compounding this, common diagnostic tools such as magnetic resonance imaging are not independently appropriate for a conclusive diagnosis of TOS. Patients with TOS can initially present acutely due to symptom exacerbations or emergent situations, necessitating multimodal diagnostic methods and early TOS recognition to improve patient outcomes, particularly in emergency department (ED) settings.

**Case Report:**

A 22-year-old male presented with chronic symptoms of numbness and weakness in his right hand in addition to chest pain that radiated into his right elbow, along with a diminished right radial pulse. The patient also suffered from acute symptomatic exacerbations of total arm asthenia, paresthesia, and what the patient described as “an intensely cold hand” during football practice. He was eventually treated with a right first-rib resection to decompress the brachial plexus, which resulted in complete symptom resolution and recovery.

**Conclusion:**

Due to the serious long-term complications associated with uncorrected brachial plexus compression and the fact that TOS patients can initially present to ED settings with acute exacerbations, it is important for emergency clinicians to be able to recognize and either treat or appropriately refer patients for treatment. The ED is equipped to enable physicians to perform a comprehensive diagnostic assessment because they often have access to the diagnostic modalities necessary for diagnosing thoracic outlet syndrome.

## INTRODUCTION

The development of thoracic outlet syndrome (TOS) can arise from anatomic variation, repetitive motions, trauma, or malignancy.^1^ Trauma and bleeding can lead to direct compressive forces on neurovascular structures followed by post-injury fibrosis that can create chronic constrictive force, causing the symptoms of TOS.^1^ Consequently, motor-vehicle collisions and midshaft clavicular fractures are common examples of underlying causes of TOS.^1^ Repetitive motions can cause muscle hypertrophy that engenders neurovascular compression particularly in patients who experience overuse injuries, resulting in insidious small hemorrhages and microfibrosis.^1^ Anatomic osseous and muscular variation cause direct compressive force on neurovascular structures, with the most notable examples being the presence of a cervical rib and congenital supernumerary scalene muscles.^1^ A cervical rib is usually an asymptomatic occurrence, but it can be a predisposition for the development of TOS following a neck trauma.^2^ Malignancy is another well-documented etiology of TOS, particularly in patients who develop superior pulmonary sulcus tumors.^1^ These examples illustrate the utility of increased awareness of TOS in the emergency department (ED) due to the common nature of these presentations to ED settings, often making emergency physicians the initial point of medical evaluation for these patients.

## CASE REPORT

A 22-year-old male presented with chronic symptoms of numbness in his right hand in addition to chest pain radiating to his right elbow and a diminished right radial pulse. The patient also suffered from acute symptomatic exacerbations of total arm asthenia, paresthesia, and what the patient described as “an intensely cold hand” during football practice. He was a lifelong overhead-throwing athlete with a pertinent surgical history of an uncomplicated superior labrum, anterior to posterior surgical repair of his right shoulder. After plain radiographs and a magnetic resonance imaging (MRI) study of the right shoulder showed an absence of abnormal findings, the patient was left without a diagnosis. His symptoms continued to progress over the course of an additional year, motivating him to seek additional evaluation at a thoracic and vascular clinic.

The additional workup included a Doppler ultrasound vascular study and in-depth physical exam. The results of the Doppler ultrasound vascular studies demonstrated decreased distal blood flow in the right arm vasculature compared to the left, and the physical examination demonstrated a positive Adson maneuver and Roos test, leading to an ultimate diagnosis of TOS. Several weeks later, he was admitted to the hospital and underwent a right first-rib resection performed by thoracic surgery to decompress the brachial plexus, specifically, the medial division leading to the ulnar nerve along with the subclavian artery and the surrounding vasculature. After the procedure, he was discharged on hospital day two and subsequently treated with six weeks of physical therapy. The patient made a full recovery, including complete restoration of range of motion and blood flow, as well as cessation of pain and numbness. Progress was monitored at yearly postoperative visits, where his vascular supply was reassessed with arterial and venous Doppler ultrasounds along with upper extremity vascular studies to ensure complete termination of symptomatology and disease pathology.

## DISCUSSION

Although the patient detailed above underwent a diagnostic process that was lengthy and included care from specialist clinics, his diagnosis was made with diagnostic studies and physical exam maneuvers that are readily available to most clinicians. A thorough understanding of TOS and the available diagnostic modalities can expedite future patient care.

Thoracic outlet syndrome was defined in 1956 to describe a spectrum of upper extremity symptoms caused by compression of the neurologic and vascular structures of the thoracic outlet.^3^ There are three points of compression in TOS. The first is at the interscalene triangle, which consists of the anterior and middle scalene muscles and the first rib.^4^ The symptomology seen from compression in this region is due to impingement of the subclavian artery or all trunks of the brachial plexus.^4^ The second point is the costoclavicular space, which is defined by the anterior border of the clavicle, subclavius muscle, the costocoracoid ligament, the posterior border by the first rib and scalene muscles, and the lateral border by the scapula.^4^ The symptomology seen from compression in this region resembles the structures described above.^4^ The third point is the retropectoral space, which is inferior to the coracoid process adjacent to the second through fourth ribs, and posterior to the pectoralis muscle.^4^ Compression in this region causes symptoms due to impingement of the axillary artery, axillary vein, and the brachial plexus cords.^4^

**CPC-EM Capsule**

What do we already know about this clinical entity?

*Thoracic outlet syndrome (TOS) can be difficult to diagnose due to its diverse presentation, which is dependent on arterial, venous, or neurogenic compression symptoms*.

What makes this presentation of disease reportable?

*A young male diagnosed with neurogenic and arterial TOS required multiple imaging studies during his diagnostic workup*.

What is the major learning point?

*The combination of physical exam maneuvers and imaging modalities in symptom-provoking positions could be the key to streamlining the diagnostic process of TOS*.

How might this improve emergency medicine practice?

*A thorough understanding of TOS and the available diagnostic modalities can expedite future patient care*.

Thoracic outlet syndrome is subcategorized based on which impinged structures are the primary cause of the patient’s symptoms.^4^ These categories are neurogenic TOS (nTOS), venous TOS (vTOS), and arterial TOS (aTOS).^4^ Neurogenic TOS comprises 90–95% of TOS cases and is more common in women.^3^,^4^ Neurogenic TOS symptomatology is determined by which brachial plexus structures are primarily compressed, with lower plexus compression of the eighth cervical and first thoracic nerves (C8-T1) resulting in neurological symptoms in an ulnar distribution, whereas upper plexus compression of fifth cervical and seventh cervical nerves (C5-C7) resulting in more widespread neurological symptoms that occur in a supraclavicular, upper thoracic, or radial nerve distribution.^4^ In addition to these non-specific neurological findings, hand coldness and color changes (Raynaud phenomenon) are often experienced, this is caused by sympathetic overactivation from brachial plexus impingement.^4^ The variety of non-specific symptoms makes nTOS diagnosis difficult.^4^

Venous thoracic outlet syndrome comprises 3–5% of TOS cases and affects young adults of both genders who do repetitive upper-arm movements. Venous thoracic outlet syndrome (otherwise known as Paget-Schröetter syndrome) is the most easily identifiable of the TOS classifications because it is characterized by drastic upper extremity swelling, indicative of subclavian vein obstruction. It can also be accompanied by cyanosis, severe pain in the upper extremity, and a feeling of arm heaviness after activity.^2^,^4^ This obstruction is mainly due to thrombotic events that can result from repetitive vessel injury, making it most common in young, physically active individuals.^2^–^4^

Arterial TOS comprises less than 1% of cases. Arterial TOS is a rare condition caused by subclavian artery compression or thrombi obstruction presenting with unilateral symptoms of digital ischemia, coldness, pallor, non-radicular pain, or paresthesias localized to the hand and rarely accompanied by cervical or upper extremity symptoms.^2^,^4^ Almost all cases of aTOS occur in patients who have a cervical rib or an anatomically unique first rib that presses on the subclavian artery.^4^ If aTOS is left untreated, aneurysm formation, embolic events, and life-threatening ischemia can occur.^4^

After obtaining a history, physical exam maneuvers should be performed to diagnose TOS. The Adson maneuver is performed with the patient seated in a chair with their arms resting at their sides. The examiner then palpates the radial pulse on the affected side, while having the patient rotate their head to the same side and take a deep breath.^2^,^5^,^6^ A positive Adson maneuver would reveal a decreased or absent radial pulse with the onset of paraesthesias.^2^,^5^ The Roos test is arm elevation for three minutes with the arm abducted to 90° and elbow flexed to 90° while the patient opens and closes his or her hand rapidly (Figure).^7^,^8^ The Roos test is positive if symptoms are reproduced.^6^ The data on sensitivity and specificity for TOS physical exam maneuvers is variable; therefore, other imaging modalities and clinical suspicion play an important role.^9^

**Image 1:**
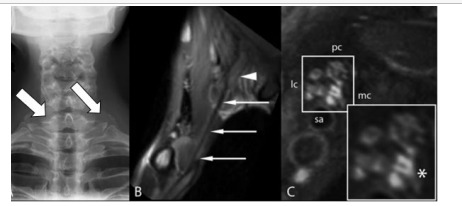
Diagnostic imaging findings for thoracic outlet syndrome: A) demonstrates a radiograph displaying bilateral cervical ribs (arrows); B) points out the fibrous band from the cervical rib to the first thoracic rib near the inferior trunk (arrows); and C) is a T2-weighted magnetic resonance image showing increased signal near the inferior trunk (asterisk). Reprinted with permission, with arrows added to the image in section A.^10^

Imaging is the next step in the diagnosis of TOS. Radiographic assessment of gross anatomical abnormalities can easily identify a cervical rib or an anomalous first rib, which raises clinical suspicion for TOS (Image 1).^2^ If a rib abnormality is not identified with a radiograph, it does not completely rule out TOS if the patient’s symptoms are highly suggestive, but it can decrease the clinical suspicion that the symptoms are being caused by bone compression on the brachial plexus structures and increase the suspicion of another cause.^2^

Following radiograph imaging, a neck and shoulder magnetic resonance imaging (MRI) study is the appropriate next step in diagnosing TOS to evaluate the brachial plexus. Magnetic resonance imaging can have an increased positive predictive value for TOS because brachial plexus compression can be caused by constricting fibrous bands and other compressed elements in the brachial plexus. These fibrous bands and compressed elements have been noted to emit increased signals in MRI images and can increase clinical suspicion.^10^ It has been shown that MRI images from TOS-positive patients demonstrate a thicker subclavius muscle and a wider retropectoralis minor space in patients with TOS compared to controls.^11^ However, TOS signs and symptoms are often not provoked in the standard anatomical position with arms positioned at the sides. Imaging in a symptom-provoking position could be a key to unlocking more usefulness of MRI in TOS diagnosis.^11^

**Image 2:**
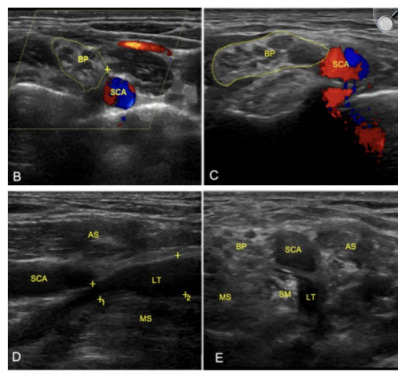
Ultrasound of the brachial plexus with and without Doppler: B) normal and C) injured side. Compare the cross-sectional view of the brachial plexus (BP) in these images. The BP is larger on the injured side. D) Longitudinal axis showing compression of the lateral trunk (LT) by the middle scalene (MS) muscle as is denoted by the measuring markers labeled 1; and E) short-axis view of the LT compression by the scalenus minimus (SM). *SCA*, subclavian artery; *AS*, anterior scalene. Reprinted with permission.^12^

Next, performing a movement-specific imaging modality is key. A Doppler ultrasound to assess vascular flow in various movements and joint articulations or a high-frequency ultrasound to assess nerve edema or compression in exacerbating positional states are the two main options.^12^ This allows the clinician to assess positional compressions and possibly identify the specific locations of compression (Image 2).

Lastly, electrodiagnostic tests such as nerve compression and conduction can be one of the most promising diagnostic tools for nTOS. Assessing the medial antebrachial cutaneous nerve in symptomatic patients with suspected TOS showed abnormal conduction velocities in action potentials.^12^ When comparing to the unaffected limb, the affected limb displayed differences in amplitudes of approximately 0.3 milliseconds. The combination of appropriate history-taking, physical exam maneuvers, and these diagnostic modalities can allow clinicians to promptly and accurately diagnose TOS, preventing the development of adverse outcomes associated with uncorrected long-term brachial plexus compression.

## CONCLUSION

Thoracic outlet syndrome is a pathology caused by compression of the brachial plexus and can cause neurologic, venous, or arterial symptoms. It can be caused by abnormal anatomic development, chronic hypertrophy, or acute events such as trauma, malignancies, or vascular thrombosis. These types of acute presentations make awareness of TOS specifically relevant to ED settings, because in these acute situations an emergency clinician is the primary point of patient evaluation. Emergency department settings are uniquely equipped to be able to mount an appropriate diagnostic plan for TOS because they often have access to the diagnostic modalities necessary for diagnosing TOS and are, therefore, able to promptly treat or refer patients to treatment, expediting patient care and preventing negative patient outcomes associated with chronic brachial plexus compression. These factors all serve to emphasize the importance of awareness and efficient diagnosis of TOS, particularly in ED settings.

## Figures and Tables

**Figure:**
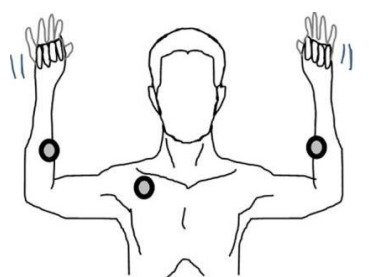
Representation of the Roos test. Circles on each arm represent regions where a diminished pulse can be felt in a positive Roos test. The circle in the right axillary region represents possible compression of the thoracic outlet. The lines around the hands exemplify rapid opening and closing of hands during the Roos test. Reprinted with permission.^8^

## References

[1] Jones MR, Prabhakar A, Viswanath O (2019). Thoracic outlet syndrome: a comprehensive review of pathophysiology, diagnosis, and treatment. Pain Ther.

[2] Sanders RJ, Hammond SL, Rao NM (2007). Diagnosis of thoracic outlet syndrome. J Vasc Surg.

[3] Peet Rm, Henrikson JD, Anderson TP (1956). Thoracic-outlet syndrome: evaluation of a therapeutic exercise program. Proc Staff Meet Mayo Clin.

[4] Kuhn JE, Lebus GF, Bible JE (2015). Thoracic outlet syndrome. J Am Acad Orthop Surg.

[5] Gillard J, Pérez-Cousin M, Hachulla É (2001). Diagnosing thoracic outlet syndrome: contribution of provocative tests, ultrasonography, electrophysiology, and helical computed tomography in 48 patients. Joint Bone Spine.

[6] Rayan GM, Jensen C (1995). Thoracic outlet syndrome: provocative examination maneuvers in a typical population. J Shoulder Elbow Surg.

[7] Demirbag D, Unlu E, Ozdemir F (2007). The relationship between magnetic resonance imaging findings and postural maneuver and physical examination tests in patients with thoracic outlet syndrome: results of a double-blind, controlled study. Arch Phys Med Rehabil.

[8] Henni S, Hersant J, Ammi M (2019). Microvascular response to the Roos test has excellent feasibility and good reliability in patients with suspected thoracic outlet syndrome. Front Physiol.

[9] Hixson KM, Horris HB, McLeod TCV (2017). The diagnostic accuracy of clinical diagnostic tests for thoracic outlet syndrome. J Sport Rehabil.

[10] Baumer P, Kele H, Kretschmer T (2013). Thoracic outlet syndrome in 3T MR neurography—fibrous bands causing discernible lesions of the lower brachial plexus. Eur Radiol.

[11] Demondion X, Bacqueville E, Paul C (2003). Thoracic outlet: assessment with MR imaging in asymptomatic and symptomatic populations. Radiology.

[12] Chen D, Gong W, Wang J (2023). Diagnosis of thoracic outlet syndrome with the lower trunk compression of brachial plexus by high-frequency ultrasonography. BMC Musculoskelet Disord.

